# Specific combination of compound heterozygous mutations in 17β-hydroxysteroid dehydrogenase type 4 (*HSD17B4*) defines a new subtype of D-bifunctional protein deficiency

**DOI:** 10.1186/1750-1172-7-90

**Published:** 2012-11-22

**Authors:** Hugh J McMillan, Thea Worthylake, Jeremy Schwartzentruber, Chloe C Gottlieb, Sarah E Lawrence, Alex MacKenzie, Chandree L Beaulieu, Petra A W Mooyer, Ronald J A Wanders, Jacek Majewski, Dennis E Bulman, Michael T Geraghty, Sacha Ferdinandusse, Kym M Boycott

**Affiliations:** 1 Children’s Hospital of Eastern Ontario Research Institute, University of Ottawa, Ottawa, ON, Canada; 2 McGill University and Genome Quebec Innovation Centre, Montréal, QC, Canada; 3 Ottawa Hospital Research Institute, University of Ottawa, Ottawa, ON, Canada; 4 Laboratory Genetic Metabolic Diseases, University of Amsterdam, Amsterdam, The Netherlands; 5 Department of Human Genetics, McGill University, Montréal, QC, Canada

**Keywords:** Polyneuropathy, Sensorineural hearing loss, Retinitis pigmentosa, Peroxisomes, Cerebellar ataxia, HSD17B4

## Abstract

**Background:**

D-bifunctional protein (DBP) deficiency is typically apparent within the first month of life with most infants demonstrating hypotonia, psychomotor delay and seizures. Few children survive beyond two years of age. Among patients with prolonged survival all demonstrate severe gross motor delay, absent language development, and severe hearing and visual impairment. DBP contains three catalytically active domains; an N-terminal dehydrogenase, a central hydratase and a C-terminal sterol carrier protein-2-like domain. Three subtypes of the disease are identified based upon the domain affected; DBP type I results from a combined deficiency of dehydrogenase and hydratase activity; DBP type II from isolated hydratase deficiency and DBP type III from isolated dehydrogenase deficiency. Here we report two brothers (16½ and 14 years old) with DBP deficiency characterized by normal early childhood followed by sensorineural hearing loss, progressive cerebellar and sensory ataxia and subclinical retinitis pigmentosa.

**Methods and results:**

Biochemical analysis revealed normal levels of plasma VLCFA, phytanic acid and pristanic acid, and normal bile acids in urine; based on these results no diagnosis was made. Exome analysis was performed using the Agilent SureSelect 50Mb All Exon Kit and the Illumina HiSeq 2000 next-generation-sequencing (NGS) platform. Compound heterozygous mutations were identified by exome sequencing and confirmed by Sanger sequencing within the dehydrogenase domain (c.101C>T; p.Ala34Val) and hydratase domain (c.1547T>C; p.Ile516Thr) of the 17β-hydroxysteroid dehydrogenase type 4 gene (*HSD17B4*). These mutations have been previously reported in patients with severe-forms of DBP deficiency, however each mutation was reported in combination with another mutation affecting the same domain. Subsequent studies in fibroblasts revealed normal VLCFA levels, normal C26:0 but reduced pristanic acid beta-oxidation activity. Both DBP hydratase and dehydrogenase activity were markedly decreased but detectable.

**Conclusions:**

We propose that the DBP phenotype seen in this family represents a distinct and novel subtype of DBP deficiency, which we have termed type IV based on the presence of a missense mutation in each of the domains of DBP resulting in markedly reduced but detectable hydratase and dehydrogenase activity of DBP. Given that the biochemical testing in plasma was normal in these patients, this is likely an underdiagnosed form of DBP deficiency.

## Background

D-bifunctional protein (DBP) deficiency is an autosomal recessive disorder of peroxisomal fatty acid oxidation. The term bifunctional originated from the discovery that this single enzyme contained multiple active domains responsible for sequential steps in peroxisomal β-oxidation. Specifically, DBP catalyzes the second step (hydration) and third step (dehydrogenation) of β-oxidation of the very long chain fatty acids (VLCFA) C26:0, branched-chain fatty acids (pristanic acid) [1] and bile acid intermediates (dihydroxycholestanoic acid (DHCA) and trihydroxycholestanoic acids (THCA)) [2]. DBP contains three domains and is encoded by the 17-β hydroxysteroid dehydrogenase type 4 (*HSD17B4)* gene. The N-terminal short-chain alcohol dehydrogenase domain is encoded by exons 1–12, the central 2-enoyl-CoA hydratase domain is encoded by exons 12–21 and the C-terminal sterol carrier protein 2-like domain (SCP-2L) is encoded by exons 21–24 [3]. DBP is a homodimeric enzyme with 79 kD subunits. After import into the peroxisome, the protein is cleaved resulting in a 35 kD dehydrogenase unit and a 45 kD hydratase plus SCP-2L unit.

DBP deficiency is classified into three subtypes depending upon the deficient activity. DBP deficiency type I is a deficiency of both 2-enyol-CoA hydratase and 3-hydroxyacyl-CoA dehydrogenase activity, DBP deficiency type II is a deficiency of hydratase activity alone, and DBP deficiency type III is a deficiency of the dehydrogenase activity alone [3]. Recent clinical and biochemical review of over 100 patients with DBP deficiency has documented a similar clinical phenotype among patients with all three biochemical subtypes [4,5]. Virtually all patients present within the first month of life with hypotonia and seizures with over two-thirds also demonstrating Zellweger-like facial features (i.e. high forehead, high arched palate, enlarged fontanelle, long philtrum, hypertelorism) [4]. Most infants (>80%) with DBP deficiency die before 2 years old, typically of respiratory complications [4]. Biochemical testing typically identifies elevated levels of plasma C26:0, DHCA, THCA as well as pristanic acid and its precursor phytanic acid [6]. Only a small minority (<2%) of DBP deficient patients will show normal biochemical testing [4,7,8]. This stresses the importance of studies in cultured skin fibroblasts in such circumstances where there is clinical suspicion of a disorder of peroxisome function.

We report two brothers with confirmed DBP deficiency. While the boys demonstrated some of the typical clinical features of peroxisome dysfunction (hearing impairment, cerebellar and sensory ataxia) they showed no demonstrable biochemical abnormality of plasma VLCFA, pristanic acid and phytanic acid or urinary bile acids. Exome sequencing was essential for detecting compound heterozygous mutations in both DBP hydratase and dehydrogenase domains Further fibroblast enzyme testing for DBP activity confirmed markedly reduced but detectable hydratase and dehydrogenase activity. We propose that our patients have a novel form of DPB deficiency, which we have designated type IV on the basis of their unique clinical, biochemical and genetic features, thereby expanding the phenotypic spectrum associated with alteration of DBP function.

## Patients and methods

Institutional research ethics board approval (Children’s Hospital of Eastern Ontario) was obtained prior to exome sequencing. Each family member provided informed consent for exome sequencing as well as permission to publish clinical information and images contained within this report.

### Patient 1

**Figure 1:**
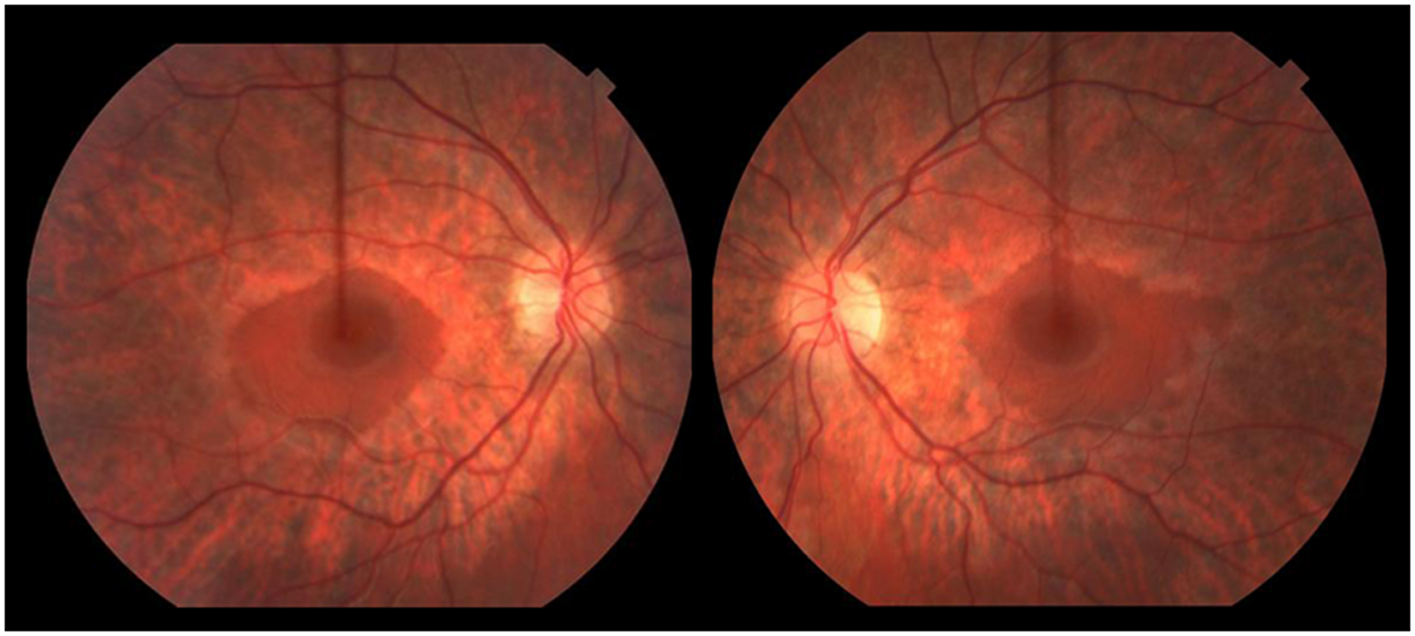
**Retinal photograph of Patient 1 at 15½ years old identified retinitis pigmentosa.** Widespread peripheral retinal atrophy was seen with relative sparing of the central macula. Clinically this corresponds to involvement of peripheral rods and relative sparing of cones.

A 16½-year-old boy was identified with expressive language delay and articulation difficulty at 3½ years old, resulting from a moderate-to-severe sensorineural hearing impairment. His language development completely normalized with hearing aids. His early gross and fine motor development was normal; he was able to skate and play hockey as a child. He did well academically within a regular classroom setting. At 11 years old, he developed insidious, progressive gait ataxia. He was found to have bilateral pes cavus and mild hammertoe foot deformity, diffuse areflexia and flexor plantar responses. Very mild weakness of anterior compartment muscles was noted prompting prescription of ankle-foot orthoses. Small fiber sensory testing was slightly decreased in a stocking distribution. Vibration sense and proprioception remained intact. Mild dyscoordination was apparent; rapid finger movements were slow and heel-to-shin testing was impaired. Romberg sign was negative. Nerve conduction studies (Table 1) revealed a mild sensorimotor polyneuropathy with demyelinating features (motor conduction velocities; 20–25 m/sec; normal upper-extremity is ≥50 m/sec and lower extremity ≥40 m/sec). Sensory and motor nerve amplitudes were normal except for low common peroneal nerve CMAP amplitude. Over time, his pes cavus worsened and his nerve conduction studies showed evidence of progressive demyelination (increasing latency prolongation) and length-dependent axonal loss (progressive loss of motor and sensory amplitudes). Fundoscopic examination at 15½ years old revealed widespread peripheral retinal atrophy with sparing of the central macula (i.e. severe rod dysfunction with relative sparing of cones) consistent with retinitis pigmentosa (Figure 1). Visual acuity was normal. Visual evoked potentials were normal. Scotopic rod electroretinograms (ERG) revealed mild abnormality, indicating rod dysfunction in the peripheral retina. Photopic cone and 30 Hz flicker were also abnormal indicating cone dysfunction. At his last clinical follow-up at 16½ years old he remained ambulatory for short distances at home but due to his significant ataxia, a wheelchair is required for longer distances. He showed no evidence of growth or pubertal delay. His height has followed the 10-25^th^ %ile and at 16-years old he was noted to have age-appropriate pubertal development; Tanner IV pubic hair and 12–15 mL testicular volume. His cognition, language and vision remain intact. He has no clinical or biochemical evidence of renal or hepatic involvement.

**Table 1:** Nerve conduction studies

	**Normal**	**Patient 1**			**Patient 2**
		**Age at study**			
		**12 years**	**13 years**	**16 years**	**13 ½ years**
**MOTOR**:					
Median nerve					
DML (wrist to APB)	< 4.2		3.6	**5.4**	3.4
CMAP (mV)	≥ 3.9		9.2	4.1	8.6
CV (m/sec)	> 47		**27**	**27**	**33**
Ulnar nerve					
DML (msec; wrist to ADM)	< 3.4	2.6	3.1	3.6	2.2
CMAP (mV)	≥ 5.9	13.1	12.7	**4.6**	7.7
CV (m/sec)	> 47	**25**	**27**	**23**	**24**
Tibial nerve					
DML (msec; ankle to AH)	< 6.0	5.3	**6.2**	**10.5**	4.5
CMAP (mV)	≥ 3.9	3.3	3.6	**0.5**	6.6
CV (m/sec)	> 39	**21**	**22**	**15**	**26**
Peroneal nerve					
DML (msec; ankle to EDB)	< 6.0	5.0		**8.5**	4.5
CMAP (mV)	≥ 2.4	**1.7**		**0.8**	2.9
CV (m/sec)	> 39	**22**		**17**	43
**SENSORY:**					
Median nerve					
PL (msec; wrist to digit-II)	< 3.2	2.5	2.8	**3.4**	2.1
SNAP (μV)	≥ 14	67	69	15	36
CV (m/sec)		**46**	**39**	**40**	52
Ulnar nerve					
PL (msec; wrist to digit-V)	< 3.3	2.0	2.2	3.1	2.2
SNAP (μV)	≥ 9	43	64	26	44
CV (m/sec)		47	50	**35**	43
Sural nerve					
PL (msec; calf to lat mall)	< 4.2	3.8	3.0	**NR**	2.7
SNAP (μV)	≥ 5	11	17	**NR**	9.5
CV (m/sec)		**42**	**40**	**NR**	**35**

Bold and underlined values are abnormal. All sensory responses are antidromic.

Legend: DML=distal onset motor latency; CMAP=compound motor action potential; CV=conduction velocity; PL=peak onset latency; APB=abductor pollicus brevis; ADM=abductor digiti minimi; AH=abductor hallucis; EDB=extensor digitorum brevis; NR=no response.

**Figure 2:**
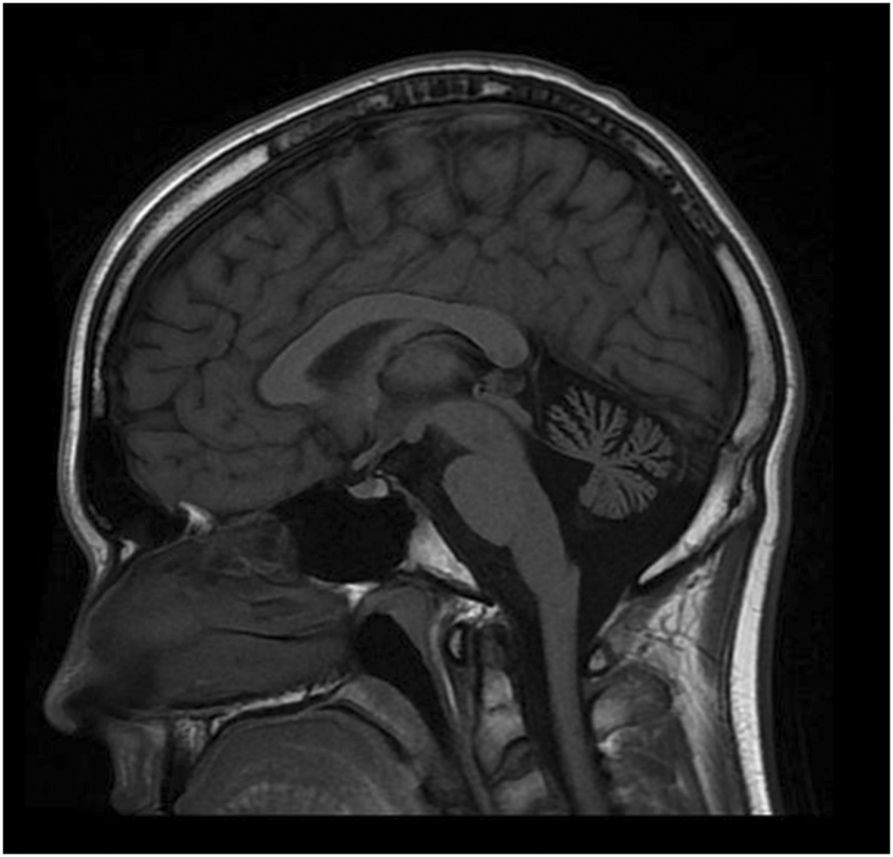
**MRI of the brain.** MRI was performed on Patient 1 at 12 years of age. T1W1 sagittal image demonstrates prominent cerebellar atrophy involving superior and middle cerebellar folia. Axial images (not shown) revealed normal subcortical and cerebellar white matter. MR spectroscopy was normal (not shown). Repeat MRI imaging at 16 years old was unchanged (not shown). MRI of the brain (Patient 2) showed a similar pattern but milder cerebellar atrophy (not shown).

MRI of the brain at 12 years of age revealed cerebellar atrophy (Figure 2). Extensive genetic and metabolic testing was performed over a span of several years, including normal *PMP22* duplication / deletion analysis, *MPZ*, *GJB1*, *PMP22* and *c10orf2* gene sequencing, spinocerebellar ataxia panel (SCA 1,2,3,6,7,8,17), Friedreich ataxia expansion testing and serum vitamin E level. Chromosome microarray was normal. Muscle biopsy (at 15-years old) revealed mild neurogenic changes. Muscle respiratory chain enzyme testing was normal. He was confirmed to have normal levels of plasma adrenal and testicular sex steroids.

Family history was remarkable for his younger brother with similar features; his parents, older sister and extended family members were all unaffected. His parents were not consanguineous, and were of German and Irish descent.

### Patient 2

The 14-year-old younger brother was identified at 2-years of age with moderate-to-severe sensorineural hearing loss requiring hearing aids. His early neurodevelopment was normal. He remains quite physically active; and at last follow-up was able to ice-skate 3 km and cross-country ski for 1-1½ hours. He does not require ankle-foot orthoses or any other assistive devices. His exam was significant for very mild pes cavus and hyporeflexia (biceps 1+, brachioradialis 1+, triceps 2+, patella 1+, ankle jerk 0), flexor plantar responses and mild anterior compartment weakness (tibialis anterior 4+/5, peroneus longus 4+, extensor hallucis longus 4). Sensory testing was normal except for pin-prick hyperesthesia to his toes. Mild ankle tightness was noted. Coordination was normal. Nerve conduction studies (Table 1) revealed a mild sensorimotor polyneuropathy with demyelinating features. Fundoscopic examination revealed widespread peripheral retinal atrophy with sparing of the central macula. Visual acuity and ERG were normal. His cognition is intact and he achieves good academic grades within a regular classroom setting. He has demonstrated a normal growth velocity although his height has remained just below the 5^th^ %ile. At almost 14 years old, he shows no sign of pubarche with Tanner I pubic hair and 5 mL testicular volume. At a chronological age of 13 years 8 months his bone age was 11 years old, consistent with constitutional delay of growth and puberty. He shows no evidence of renal or hepatic involvement.

### Exome sequencing

Exome capture and high-throughput sequencing of DNA from the two brothers was performed at McGill University and Genome Québec Innovation Centre (Montréal, Canada). Total genomic DNA was extracted from blood following standard procedures. Exome target enrichment was performed using the Agilent SureSelect 50Mb All Exon Kit, and sequencing (Illumina HiSeq) generated 65 Gbp of 100 bp paired-end reads per sample. Mean coverage of coding sequence regions (CCDS), after accounting for duplicate reads was 237x and 212x for each of the affected brothers. 91.5% of CCDS bases in Patient 1 and 90.0% of CCDS bases in Patient 2 had ≥20x coverage and ≥5x coverage was seen in 96.8% of CCDS bases in both siblings. An in-house annotation pipeline was used to call and annotate coding and splice-site variants. Reads were trimmed and sequences with a matching (opposite) read were aligned to hg19 using BWA [9]. Duplicate reads were marked using Picard [10] and excluded. Single nucleotide variants and short insertions and deletions (indels) were called using SAMtools pileup [11] and varFilter and quality-filtered to require a minimum 20% of reads supporting the variant call. Variants were annotated using Annovar [12] as well as custom scripts to select coding and splice-site variants, and to exclude common (≥1% minor allele frequency) polymorphisms represented in the NHLBI exome server [13], or in 435 control exomes sequenced at our center. Variants were prioritized based on those identified in both affected patients. Given the presumed autosomal recessive mode of inheritance, only genes with homozygous or multiple heterozygous variants were considered.

### Variant validation

Sanger sequencing was used to validate mutations identified by next-generation sequencing and to evaluate segregation of variants in the family. Blood samples were obtained and DNA was extracted from the affected brothers and unaffected parents and sister. PCR was performed with primers 5^′^-GAGTGGATAGGTTGAGAATGTCAGTG-3^′^ and 5^′^-TTTAGACAGACAGCCTTAGTCGGG-3^′^ to test for the c.101C>T variant and 5^′^-ACCAATAACCAGCCATGTTTCCT-3^′^ and 5^′^-TCCTACCTTTCCATATCCTTTGCAT-3^′^ to test for c.1547T>C variant.

### Biochemical analysis

Biochemical testing in fibroblasts was performed as described [4] (Table 2) .

**Table 2:** Patient plasma and fibroblast biochemical analyses

**Test**	**Units**	**Patient 1**	**Patient 2**	**Reference range**
**Plasma:**				
**VLCFA concentration**				
C26:0 Hexacosanoic	μg/mL	0.220	0.270	0.23 ± 0.09*
C26/C22		0.021	0.012	0.01 ± 0.004*
C24/C22		0.918	0.967	0.84 ± 0.918*
Phytanic acid	μg/mL	1.260	0.810	< 3.0
Pristanic acid	μg/mL	0.140	0.060	< 0.3
**Fibroblasts:**				
**Catalase immunofluorescence**		Near-normal	Normal	Normal
**VLCFA concentration:**				
C26:0 concentration	μmol / g protein	0.20	0.20	0.18 - 0.38
C24:0 concentration	μmol / g protein	6.52	6.79	7.76 - 17.66
C22:0 concentration	μmol / g protein	3.00	3.23	3.84 - 10.20
Ratio C26:0 / C 22:0		0.07	0.06	0.03 - 0.07
Ratio C24:0 / C 22:0		2.18	2.10	1.55 - 2.30
**Peroxisome function:**				
β-oxidation (of C16:0)	pmol / (mg protein / hour)	3876	5061	3330 - 7790
β-oxidation (of C26:0)	pmol / (mg protein / hour)	1290	1325	800 - 2040
β-oxidation (of pristanic acid)	pmol / (mg protein / hour)	**157**	**248**	790 - 1690
α-oxidation (of phytanic acid)	pmol / (mg protein / hour)	38	45	28 - 95
**D-bifunctional protein activity:**				
Hydratase	pmol / (mg protein / min)	**43**	**53**	115 - 600
Dehydrogenase	pmol / (mg protein / min)	**2**	**3**	25 - 300
**Immunoblotting:**				
DBP 79 kDa		**Trace**	**Trace**	Present
DBP 45 kDa		**Absent**	**Absent**	Present
DBP 35 kDa		**±/−**	**±/−**	Present

Plasma testing was performed at Kennedy-Krieger Institute (Baltimore, USA). Fibroblast testing was performed at the Laboratory Genetic Metabolic Diseases (Amsterdam, The Netherlands).

Bold and underlined values are abnormal; *Indicates mean +/− 1 standard deviation.

## Results

### Exome sequencing and variant validation

**Figure 3:**
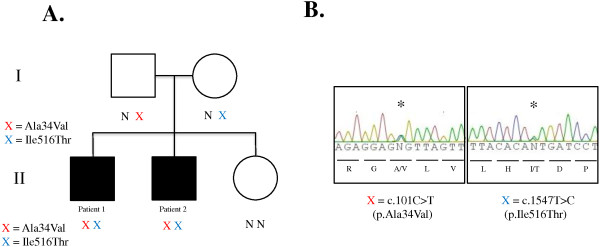
**Sanger sequencing and segregation.** (**A**) Pedigree of family with DBP deficiency. *X=*variant; *N=*normal. (**B**) Sanger sequencing validation of *HSD17B4* variants identified by exome sequencing. Genomic DNA was amplified for sequencing with primers flanking exon 2 and 18 (see text for primer sequences). Asterisks indicate heterozygous mutations. Red X = c.101C>T (p. Ala34Val) at chr5:118792052. Blue X = c.1547T>C (p. Ile516Thr) at chr5:118860954.

Two variants, c.1547T>C (p.Ile516Thr) at chr5:118860954 and c.101C>T (p.Ala34Val) at chr5:118792052 were identified in *HSD17B4* (Table 3). c.1547T>C was found in a region of very low coverage, present in only 2 of 8 reads (variant quality of 18.4%) in Patient 2 and therefore was only identified by visual inspection once the two variants had been annotated in his brother. Sanger sequencing confirmed c.1547T>C and c.101C>T in both brothers. Parents were each heterozygous for one of the mutations; c.1547T>C; p.Ile516Thr inherited from the mother and c.101C>T; p.Ala34Val inherited from the father (Figure 3). Both mutations were predicted to be deleterious by the SIFT algorithm [14] (SIFT score 0.03 and 0 respectively) and probably damaging by PolyPhen2 [15] (HumDiv score 0.999 and 0.997 respectively). Both mutations have been reported separately to be disease-causing [5]; however, they have not been seen in combination before.

**Table 3:** Filtering of exome sequencing variants

Genes with missense, nonsense, indel or splice variants	6453
Genes with rare mutations^1^	372
Genes with mutations shared by siblings	109
Genes with homozygous/ multiple heterozygous mutations	2^2^

^1^Variants were filtered out if they had a minor allele frequency (MAF) >0.01 in the 1000 genomes database, the NHLBI exome server or were seen in >2 of 435 control exomes sequenced at our center.

^2^The 2 genes were *C16orf82* and *FAM83C*; these genes were rejected as candidates since two control samples possessed identical sequence variants in *C16orf82* and one control sample possessed an identical sequence variant in *FAM83C*. The 109 genes with at least one mutation shared by the siblings were then re-examined and the *HSD17B4* mutations were identified. The second *HSD17B4* mutation was not initially identified in Patient 2 due to low coverage (given presence in only 2/8 reads it was given a variant quality of 18.4 which is below our threshold of 20).

### Biochemical analyses

Plasma levels of VLCFA and branched chain fatty acids (pristanic acid and phytanic acid) were normal (Kennedy Krieger Institute, Baltimore, USA; Table 2). Plasma docosohexanoic acid (DHA) levels were also normal (data not shown, Kennedy Krieger Institute, Baltimore, USA). Urine was analyzed using fast atom bombardment ionization mass spectrometry with no abnormalities identified in urine bile acid secretion (data not shown, Cincinnati Children’s Hospital Medical Center, USA). Fibroblast studies at the Laboratory of Genetic Metabolic Diseases (Academic Medical Center, Amsterdam, The Netherlands) revealed normal VLCFA levels and normal C26:0 beta-oxidation, but reduced pristanic acid beta-oxidation activity (Table 2). Catalase immunofluorescence studies showed normal to near-normal peroxisomal staining with respect to number and morphology of peroxisomes (Table 2). Peroxisomes were slightly increased in size in Patient 1. DBP enzyme activity measurements revealed reduced hydratase and dehydrogenase activities (Table 2). The amount of DBP protein was reduced on immunoblot (Table 2 and data not shown, Laboratory of Genetic Metabolic Diseases, Academic Medical Center, Amsterdam, The Netherlands).

## Discussion

Our exome sequencing of two siblings with a previously undiagnosed neurodegenerative disorder has detected compound heterozygous mutations c.101C>T (p.Ala34Val) and c.1547T>C (p.Ile516Thr) in *HSD17B4* affecting the dehydrogenase and hydratase domains, respectively. Both missense mutations have been previously reported, but in both cases with a second missense mutation affecting the identical DBP domain on the other allele [5]. The first case was compound heterozygous for p.Ala34Val and p.Phe237Ser; both mutations affecting the dehydrogenase domain resulting in DBP type III and an isolated dehydrogenase deficiency. The second case was compound heterozygous for p.Ile516Thr and p.Asn457Tyr; in this instance both mutations occur within the hydratase domain causing DBP type II and an isolated hydratase deficiency. Although this patient with DBP type II survived >13.5 years, cognitive and language deficits were significant.

An obvious explanation for the relatively milder clinical phenotype observed in our patients is the fact that only one domain on each allele is affected with a less severe mutation. The attenuated clinical phenotype and biochemical testing indicates normal transcription, translation, and the normal import of the DBP enzyme into an intact and functional peroxisome. The latter is supported by normal catalase immunoflorescence observed in our patients indicating normal peroxisome biogenesis and morphology; a finding not seen in patients with the more severe form of DBP deficiency [4]. Little information is available on the impact of the p.Ala34Val mutation and analysis demonstrated low residual enzyme activity of the dehydrogenase domain. The p.Ile516Thr is located at the dimerization interface of the hydratase subunits but does not abolish dimerization completely, implying residual activity [5]. The apparent discrepancy between the residual hydratase activity (~40% of lower limit of normal) and absence of the hydratase domain on immunoblot may in part be accounted for by the fact that L-Bifunctional protein (L-BP) is responsible for part of the measured hydratase activity because it can metabolize the substrate THC:1-CoA to the (24*S*,25*S*)-isomer of 24OH-THC-CoA which cannot be metabolized further by the dehydrogenase domain of L-BP. The result could also, in part, represent a combination of altered structure and stability of the mutant DBP enzyme. Finally, both sets of homodimers (hydratase and dehydrogenase domains) likely have some physical and functional relationship to each other. A mutation in one domain has the potential to alter function and stability of the other unit even if a mutated domain (e.g. dehydrogenase) forms a dimer with an adjacent wild-type domain (e.g. hydratase). As such, the in vivo function of this enzyme appears more complicated than that predicted by in vitro studies.

Although some other peroxisome diseases resulting from single enzyme defects can present in adulthood (i.e. acyl-CoA oxidase (ACOX) deficiency [16] and sterol carrier protein X (SCPx) deficiency [17]), this has not been reported for DBP deficiency [4]. Since both hydratase and dehydrogenase activities are affected, our patients would be deemed to be type I under the current DBP deficiency classification. However, the significant majority of type I-deficient patients have mutations in *HSD17B4* encoding truncated or unstable proteins resulting in a severe phenotype and poor survival [4]. In our estimation, the mild clinical and biochemical phenotype in our patients warrant a new classification. We therefore propose a novel variant of DBP deficiency, designated DBP type IV, due to compound heterozygous mutations affecting two different domains of DBP but associated with a relatively milder clinical and biochemical phenotype.

Our newly proposed subtype of DBP deficiency (type IV) would also apply to two sisters recently diagnosed with Perrault syndrome caused by compound heterozygous mutations within *HSD17B4,* one affecting the dehydrogenase domain and one the hydratase domain, similar to our patients. In this instance, the sisters’ relatively milder phenotype was characterized by sensorineural deafness, mild intellectual disability, sensorimotor polyneuropathy, short stature and ovarian dysgenesis [7]. Exome sequencing was also essential in obtaining this diagnosis but complete biochemical testing including DBP enzyme activity measurement was not reported. Our patients differ from these sisters by their normal intelligence and pubertal development (in the older brother).

The overall incidence of peroxisomal disorders is approximately 1 in 5,000 newborns [6], most of these cases are severe and are thus readily ascertained. In striking contrast to previously reported patients [4,5], the two brothers described here did not demonstrate any neonatal or infantile symptoms, moreover they continue to demonstrate normal cognition. Their slow clinical course of DBP deficiency has allowed, for the first time, serial electrodiagnostic testing in DBP patients; the clinical exams and nerve conduction studies spanning several years (Table 1) document a gradual decline of coordination reflecting increasing cerebellar and sensory nerve dysfunction. The progressive sensorimotor polyneuropathy demonstrated uniform conduction velocity slowing, reminiscent of that seen with many hereditary demyelinating polyneuropathies (e.g. Charcot-Marie-Tooth, type 1). The older sibling (Patient 1) not only demonstrated progressive demyelination (increasing latencies and conduction velocity slowing) but also evidence of progressive, length-dependent axonal loss. The introduction of readily available exome sequencing into rare disease clinics will lead to the recognition of additional patients with milder variants of DBP deficiency and will improve our understanding of the phenotypic spectrum and natural history of this and other diseases.

## Conclusion

We propose that the DBP phenotype seen in this family represents a distinct and novel subtype of DBP deficiency, which we have termed type IV based on the presence of a missense mutation in each of the domains of DBP, reduced but detectable hydratase and dehydrogenase activities and relatively milder clinical and biochemical features. Without exome sequencing, the diagnosis of DBP deficiency may not have been made in our patients given their normal biochemical testing in plasma and urine. These patients highlight the importance of exome sequencing as a diagnostic tool, particularly in phenotypically and genotypically heterogeneous disorders or in cases with atypical clinical presentations.

## Abbreviations

DBP: D-bifunctional protein; HSD17B4: Hydroxysteroid 17-β dehydrogenase 4; SIFT: Sorting Intolerant From Tolerant; PolyPhen-2: Polymorphism Phenotyping; VLCFA: Very long-chain fatty acid.

## Competing interests

The authors declare that they have no competing interests.

## Authors' contributions

HJM, TW and KMB wrote the manuscript. KMB, DB and AM designed and coordinated the study. HJM, CCG, SEL, MTG and KMB provided subspecialist consultation services, serial clinical examinations and diagnostic testing. JS and JM carried out the analysis of the next-generation sequencing data. CLB, TW and KMB contributed to the collection of clinical data, informed consent and arranged for Sanger sequencing of *HSD17B4* gene. PAWM, RJAW and SF carried out biochemical testing on fibroblasts. All authors read and approved the final manuscript.
